# Presence of ESBL/AmpC -Producing *Escherichia coli* in the Broiler Production Pyramid: A Descriptive Study

**DOI:** 10.1371/journal.pone.0079005

**Published:** 2013-11-07

**Authors:** Cindy M. Dierikx, Jeanet A. van der Goot, Hilde E. Smith, Arie Kant, Dik J. Mevius

**Affiliations:** 1 Department of Bacteriology and TSEs, Central Veterinary Institute (CVI), part of Wageningen UR, Lelystad, The Netherlands; 2 Department of Epidemiology Crisis Organization and Diagnostics, Central Veterinary Institute (CVI), part of Wageningen UR, Lelystad, The Netherlands; 3 Department of Infection Biology, Central Veterinary Institute (CVI), part of Wageningen UR, Lelystad, The Netherlands; 4 Utrecht University, Faculty of Veterinary Medicine, Utrecht, The Netherlands; Institut National de la Recherche Agronomique, France

## Abstract

Broilers and broiler meat products are highly contaminated with extended spectrum beta-lactamase (ESBL) or plasmid-mediated AmpC beta-lactamase producing *Escherichia coli* and are considered to be a source for human infections. Both horizontal and vertical transmission might play a role in the presence of these strains in broilers. As not much is known about the presence of these strains in the whole production pyramid, the epidemiology of ESBL/AmpC-producing *E. coli* in the Dutch broiler production pyramid was examined. Cloacal swabs of Grandparent stock (GPS) birds (one−/two-days (breed A and B), 18 and 31 weeks old (breed A)), one-day old Parent stock birds (breed A and B) and broiler chickens of increasing age (breed A) were selectively cultured to detect ESBL/AmpC-producing isolates. ESBL/AmpC-producing isolates were found at all levels in the broiler production pyramid in both broiler breeds examined. Prevalence was already relatively high at the top of the broiler production pyramid. At broiler farms ESBL/AmpC producing *E. coli* were still present in the environment of the poultry house after cleaning and disinfection. Feed samples taken in the poultry house also became contaminated with ESBL/AmpC producing *E. coli* after one or more production weeks. The prevalence of ESBL/AmpC-positive birds at broiler farms increased within the first week from 0–24% to 96–100% independent of the use of antibiotics and stayed 100% until slaughter. In GPS breed A, prevalence at 2 days, 18 weeks and 31 weeks stayed below 50% except when beta-lactam antibiotics were administered. In that case prevalence increased to 100%. Interventions minimizing ESBL/AmpC contamination in broilers should focus on preventing horizontal and vertical spread, especially in relation to broiler production farms.

## Introduction

Infections with Extended Spectrum Beta-Lactamase (ESBL) or plasmid-mediated AmpC beta-lactamase producing *Enterobacteriaceae* are found increasingly in humans [1]. These isolates are resistant to an important group of antibiotics: the beta-lactam antibiotics. These antibiotics include penicillins and the newer generation cephalosporins, like 3^rd^ and 4^th^ generation cephalosporins. ESBL enzymes differ slightly from AmpC enzymes in their ability to hydrolyze the different beta-lactam antibiotics. ESBLs hydrolyze 3^rd^, 4^th^ generation cephalosporins and monobactams and are inhibited by clavulanic acid and cephamycins, like cefoxitin. There are multiple gene families encoding ESBLs. Genes belonging to the TEM, SHV and CTX-M families are most predominant [2]. Over the last years especially genes belonging to the CTX-M family are emerging rapidly worldwide [2]. AmpC beta-lactamases hydrolyze 3^rd^ generation cephalosporins and cephamycins, but not the 4^th^ generation cephalosporins and they are not inhibited by clavulanic acid. The most important gene-families encoding AmpC beta-lactamases are CMY, ACC, DHA and FOX, with CMY being the most predominant family and *bla*_CMY-2_ the most predominant gene [3]. During the last decade infections with ESBL or AmpC-producing bacteria have not exclusively been confined to the hospital, but community onset of infections with these organisms is also increasing [4]. Although the source of the colonization of ESBL/AmpC producing bacteria in humans is not completely understood, circumstantial evidence points also to a food-borne source [5]. When uptake of these isolates takes place (through consumption or handling of contaminated food), these isolates are able to share their ESBL/AmpC genes with other bacteria in the gastro-intestinal tract by plasmid-transfer, especially when selecting compounds, like beta-lactam antibiotics are administered [6]. ESBL/AmpC producing bacteria in the gastro-intestinal tract can act as a source for infections in other parts of the body, like the urinary tract [7]. Treatment efficacy may be impaired due to the multi-drug resistant features often found in these organisms [1].

ESBL/AmpC-producing isolates can be found in nearly all food-producing animals [8], on all kinds of meats sold at retail [9], [10], [11] and in vegetables [12]. A high prevalence of ESBL/AmpC producing isolates is found in broilers and on broiler meat [9], [13], [14], [15]. The isolates found in broilers and broiler meat carry similar ESBL-genes (mainly *bla*_CTX-M-1_ and *bla*_TEM-52_) as found in clinical isolates in humans [10], [11], moreover ESBL-genes in broiler isolates are found on similar plasmids (mainly incI1) as in human clinical isolates [10]. This suggests that contamination of broilers and broiler meat with ESBL/AmpC producing isolates can lead to human colonization and as a result to human infection with ESBL/AmpC producing pathogens.

A high prevalence of broilers shedding ESBL/AmpC producing *E. coli* is described at broiler farms in the Netherlands, Belgium and Spain [13], [14], [15].The broiler industry has a pyramidal structure in which Pedigree chickens and Great Grandparent Stock (GGPS) on the top through breeding chickens (Grandparent Stock (GPS) and Parent Stock (GP)) produce the broiler chickens on the bottom of the pyramid. Earlier studies have implicated a vertical transmission of *E. coli* isolates from broiler breeding chickens to their offspring [16], [17], [18]. Although much is known about the prevalence at broiler level, little is known about prevalence and characteristics of ESBL/AmpC-producing *E. coli* higher in the broiler production pyramid. In the Netherlands almost all levels of the broiler production pyramid are present (GPS, PS and broilers). Therefore the purpose of this study was to investigate the presence and distribution of ESBL/AmpC producing isolates in all levels of the broiler production pyramid. Moreover in a longitudinal study at the level of GPS broilers and at three broiler production farms the dynamics of ESBL/AmpC producing *E. coli* among the broiler chickens was examined.

## Materials and Methods

### One- or Two-day(s) Old GPS Chickens

GPS chickens were sampled from broiler breed A and B, which are by far the two dominant broiler production breeds globally. GPS chickens of broiler breed A were sampled in July 2009 at two days of age. These GPS chickens were imported from the UK and, as is routinely done by the breeding company for every batch of imported GPS chickens, checked for diseases by euthanizing 10 animals per farm. The breeding company agreed that we used caecal material obtained at autopsy for the detection of ESBL/AmpC producing *E. coli*. In total 80 GPS chickens (10 per production farm (n = 8)) were investigated. Fresh meconium droppings taken from fresh broiler paper from one-day old GPS chickens from broiler breed B were collected in September 2010 by the producer at the hatchery in the Netherlands. These broilers were the offspring from GGPS birds held at farms located in the UK and Ireland. In total 125 meconium samples (25 per production farm of which the GPS chickens originated (n = 5)) were collected in individual falcon tubes and transported to the laboratory. According to the information given by primary breeding company A and B the parents of the sampled GPS chickens had not received any antibiotic prior to sampling.

### Longitudinal Study in GPS of Breed A

**Figure 1:**
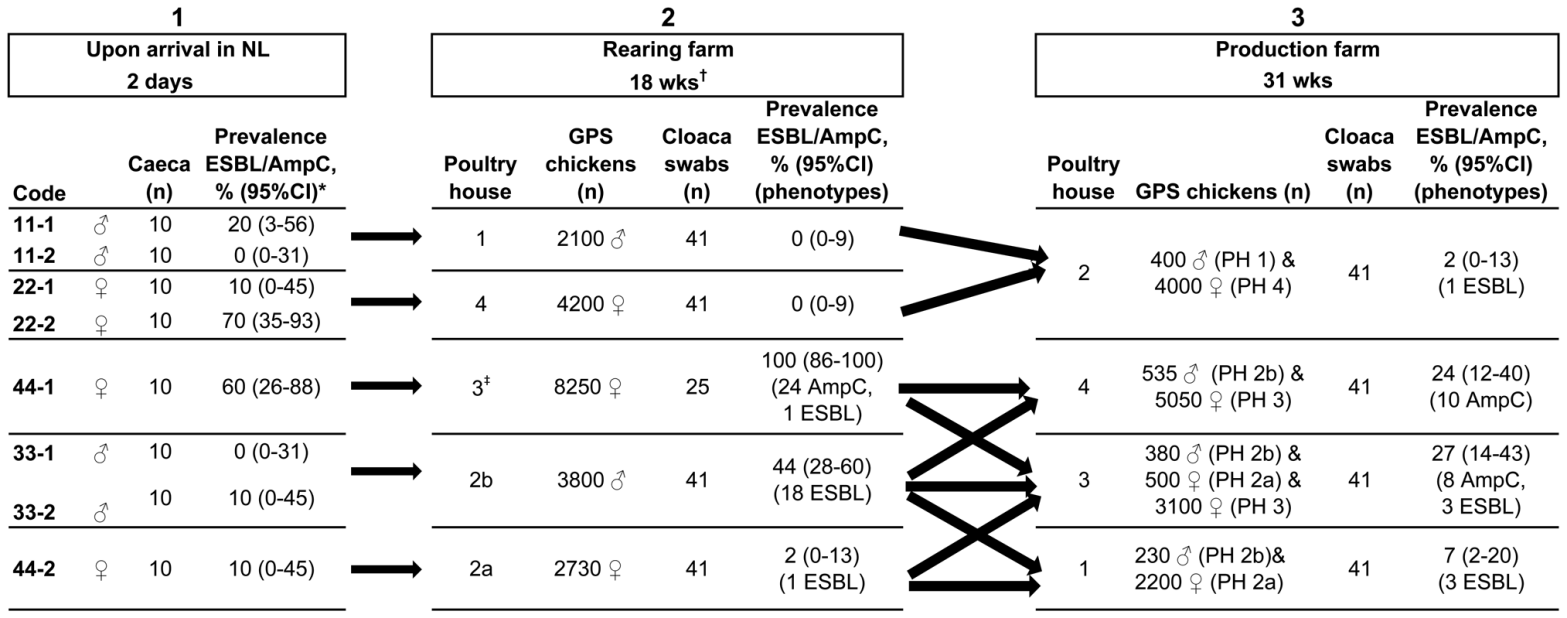
Schematic view of the prevalence of ESBL/AmpC producing *E. coli* in the broiler production pyramid. The direction of the arrows shows how the chickens were mixed at the farms. *All isolates derived from caeca collected from two-day old GPS chickens had the AmpC phenotype. ^†^All GPS chickens received enrofloxacin at arrival on the rearing farm. ^‡^These Grandparent Stock (GPS) chickens were also treated with amoxicillin-trihydrate and phenoxymethylpenicillin at respectively 2 and 8 weeks of age PH = Poultry house.

The batch of GPS chickens from broiler breed A in which 10 animals were euthanized was also sampled with consent of the farmers and the breeding company at the GPS rearing farm at 18 weeks of age (November 2009) and at the GPS production farm at 31 weeks of age (February 2010). The animals were mixed as indicated by the arrows shown in Figure 1. At arrival on the rearing farm, all chickens received enrofloxacin for three days. At two and eight weeks of age, the chickens in poultry house 3 (PH3) at the rearing farm were orally treated with amoxicillin-trihydrate and phenoxymethylpenicillin respectively. From forty-one GPS chickens per poultry house, faecal samples were collected by cloacal swabs, resulting in 205 and 164 samples from GPS chickens at rearing and production farm, respectively. The first 25 samples were processed directly in the laboratory and when not all samples were positive, the remaining 16 samples (kept in 1 mL of buffered peptone water supplemented with 30% glycerol by −20°C) were also analyzed for the presence of ESBL/AmpC producing *E. coli*. In the end, a total of 189 and 164 samples, respectively from GPS rearing and production farms were analyzed.

### One-day Old PS Chickens

At the PS hatcheries of broiler breed A and B, fresh meconium droppings of one-day old PS chickens were taken from fresh broiler papers and collected in individual plastic containers in the hatchery. In that way a minimum of 25 PS chickens per farm of origin was sampled. This was done with consent of the breeding companies. If the eggs from one farm of origin were divided over more than one hatching unit, 25 PS chickens from each of those hatching units were sampled. This resulted in a maximum of 100 samples per farm of origin. From broiler breed A in October 2009, the eggs were derived from nine different production farms. These eggs were divided over 21 hatching units. This resulted in 649 meconium samples (one sample was lost before analysis) that were analyzed for the presence of ESBL/AmpC producing *E. coli*. From broiler breed B in March 2010, the eggs derived from eight farms of origin, were divided over six hatching units. This resulted in 325 meconium samples that were analyzed for the presence of ESBL/AmpC producing *E. coli*. At both hatcheries the environment of the hatching unit (after hatching, before cleaning) was also sampled. This was done by taking five individual environmental swabs per hatching unit.Some of the parents of the PS chickens of breed B had received tylosin or enrofloxacin and some of the parents of PS chickens of breed A had received either amoxicillin, doxycycline and tilmicosin (as two separate treatments), amoxicillin combined with sulfaclozine or amoxicillin and sulfaclozine (as two separate treatments) within three months before sampling.

### One-day Old Broiler Chickens

At the broiler hatchery broiler chickens are produced. Although this hatchery hatches eggs of both broiler breeds A and B, only eggs from broiler breed A were hatched at the sampling day in February 2010. Again 25 chickens per farm of origin were sampled, the same way as done at the parent hatcheries and with consent of the hatchery. The eggs were derived from twelve different production farms divided over sixteen hatching units. This resulted in 425 meconium samples tested for the presence of ESBL/AmpC producing *E. coli*. The environment was sampled as described for the PS hatcheries. No information about antibiotic treatments of the parents of the broilers was available.

### Longitudinal Study at Broiler Farms

Three broiler farms were included (Farm X, Y and Z) in a longitudinal study on ESBL/AmpC prevalence at these farms. With consent of the farmers, from October 2010 through January 2011 in total four poultry houses on these three farms were visited (X-1, Y-1, Y-2 and Z-1). The broiler farms were visited at day −1 (when the broilers had not yet arrived), day 0 (the day the animals arrived in the poultry house), week 1, 2, 3, 4 and 5. At day −1, the environment per poultry house was sampled by the researcher according to Dutch regulations for *Salmonella* control. Briefly, swabs were taken from the floor (n = 25), drinking system (n = 16), feeding system (n = 12), valves at the air inlet (n = 5), the floor of the feed compartment (n = 1), one boot of the farmer (n = 1), and one sample was taken from the bedding material. All swabs were individually processed in the lab with the exception of the swabs taken in poultry house X-1. These were pooled per sample place with a maximum of five swabs in each pool. At week one to five, 25 cloacal swabs per poultry house were taken and on every visit one sample per poultry house was taken from the feed that was present inside the poultry houses. For poultry houses Y-1, Y-2 and Z-1, also feed samples taken outside the poultry house in week five, week five and week four, respectively, were analysed. The samples were phenotypically analysed for the presence of ESBL/AmpC producing *E. coli* and the isolates were not genetically characterized.

### Microbiological Analysis of Samples

All faecal samples were spread on MacConkey agar (Becton Dickinson) supplemented with 1 mg/L cefotaxime (with and without aerobic pre-enrichment with Luria-Bertani broth (Becton Dickinson) containing 1 mg/L cefotaxime) and incubated aerobically overnight at 37°C. All environmental, bedding and feed samples were cultured on MacConkey agar with 1 mg/L cefotaxime (MacConkey^+^) after selective pre-enrichment. All morphologically typical *E. coli* colonies on MacConkey^+^ were confirmed as *E. coli* by indole test (tryptophan hydrolysis) and if negative by *E. coli* PCR [19]. One confirmed *E. coli*-type colony per sample was examined for ESBL or AmpC production by combination disc diffusion test containing cefotaxime and ceftazidime with and without clavulanic acid and cefoxitin as described [20]. The presence of ESBL/AmpC genes was determined for one isolate per phenotype per group of samples. These isolates were screened for ESBL/AmpC genes by miniaturized microarray (Alere, ATR0503). The presence of ESBL/AmpC genes was confirmed by PCR and sequencing as described [20]. To improve sequencing results a new forward primer CMY-Fseq_838_ 5′-TGG CGT ATT GGC GAT ATG TA-3′ and reverse primer CMY-Rseq_857_ 5′-TAC-ATA-TCG-CCA-ATA-CGC-CA-3′ were used.

### Susceptibility Testing

Minimum Inhibitory Concentrations (MICs) in mg/L were determined for a panel of antibiotics (ampicillin (AMP), cefotaxime (FOT), ceftazidime (TAZ), gentamicin (GEN), tetracycline (TET), sulfamethoxazole (SMX), trimethoprim (TMP), ciprofloxacin (CIP), nalidixic acid (NAL), chloramphenicol (CHL), florfenicol (FFN), streptomycin (STR), kanamycin (KAN) and colistin (COL)) by broth microdilution using the Sensititre system as described earlier [20]. Wild-type susceptibility was distinguished from non-wild type susceptibility using epidemiological cut-off values according to EUCAST (ref. www.eucast.org).

### Statistics

The ninety-five percent confidence intervals (95% CI) are given as Clopper Pearson confidence intervals using Genstat (14^th^ Edition, VSN International).

### Ethics Statement

The faecal samples derived from the birds were all taken from broilers held at farms (grandparent rearing (breed A), grandparent production (breed A) and broiler production farms (farm X, Y and Z) or in routinely process at the hatchery (grandparent breed B, parent breed A, parent breed B and broiler breed A hatcheries). The farms and hatcheries were named in this way, to respect their privacy. No animal experiment was conducted. Sampling with cloacal swabs is considered a normal diagnostic sampling method with a minimal impact on animal welfare. Meconium samples were collected as fresh droppings on fresh broiler paper at hatcheries. The samples related to ESBL-prevalence at two-days old Grandparents of breed A were collected from animals that were sacrificed (with CO2) by the breeding company for other purposes. Faecal samples taken from these animals were part of standard screening by the breeding company and these samples became available for the research described in this manuscript. Therefore approval by the Animal Care and Use Committee of the Central Veterinary Institute was not obtained. Swabs from the environment in the hatcheries and the environment of the broiler farms were taken by the researcher.

## Results

### One or Two-day(s) Old GPS Chickens

The mean prevalence of ESBL/AmpC-producing *E. coli* in GPS chickens of broiler breed A was 23% (Table 1). Prevalence at the production farms ranged from 0% (95% CI 0–31%) to 70% (95% CI 35–93%) (Figure 1.1).

**Table 1:** Summary of prevalence and characteristics of ESBL/AmpC enzymes found in the Dutch broiler production chain between June 2009 and December 2010.

Animals sampled	Year of sampling	Broilerbreed	Environmentalsamples (no. ESBL/AmpC positive/no.hatching unit)	Total no.of chickensamples	No. of farms of origin/no. of poultry houses(samples analysed perfarm/poultry house)	No. ofchickensamplesfoundpositive	MeanPrevalencein chickens(range)	No. ofChickenIsolateMolecularcharacterized	ESBL/AmpC-genes inchickensamples*	ESBL/AmpC-genes inenvironmentalsamples†
Two days old GPS	2009	A	–	80	8 (10)	18	23 (0–70)	6	*bla*_CMY-2_ (n = 6)	
One day old GPS	2010	B	–	125	5 (25)	55	44 (36–60)	5	*bla*_CMY-2_ (n = 5)	–
GPS 18 weeks	2009	A	–	189	5 (25/41)	44	29 (0–100)	4	*bla*_TEM-52c_ (n = 3),*bla*_CMY-2_ (n = 1)	–
GPS 31 weeks	2010	A	–	164	4 (41)	25	15 (2 -27)	5	*bla*_TEM-52c_ (n = 2),*bla*_TEM-52_ (n = 1),*bla*_CMY-2_ (n = 2)	–
One-day old PS	2009	A	5×5 pooledenvironmental swabsper hatchingunits (0/21)	649	9 (25/50/75/100)	2	0.3 (0–4)	2	*bla*_CTX-M-2_ (n = 1),*bla*_CTX-M-1_ (n = 1)	–
	2010	B	Five environmentalswabs per hatchingunit (6/6,6 isolatescharacterized)	325	8 (25/50/75/100)	19	5.8 (0–20)	7	*bla*_CMY-2_ (n = 5),*ampC* type 40(n = 2)	*bla*_CMY-2_ (n = 4)
One-day old broilers	2010	A	Five environmentalswabs per hatchingunit (12/16,14 isolatescharacterized)	425	12 (25/75)	8	1.9 (0–20)	3	*bla*_CMY-2_ (n = 3),	*bla*_CMY-2_ (n = 8),,*bla*_TEM-20_ ‡(n = 2),*bla*_CTX-M-1_ (n = 2),*bla*_SHV-12 _ *ampC*type 3 (n = 1)

GPS =  Grandparent Stock, PS =  Parent Stock. More details about the data displayed in grey are given in Figure 1.

* With the primers used to sequence *bla*_CTX-M-2_ no distinction can be made between *bla*_CTX-M-2_ and *bla*_CTX-M-97._

† With the primers used to sequence *bla*_SHV-12 _no distinction can be made between *bla*_SHV-12_ and *bla*_SHV-129_.

‡ *bla*_TEM-20_ with silent mutations +144G->A, +480C->T and +723A->G compared to reference sequence *bla*_TEM-20_ Y17581 (nucleotide position according to amino acid count at www.lahey/studies.org).

For broiler breed B the mean prevalence in GPS chickens was 44% (Table 1). Prevalence at the production farm ranged from 36% (95% CI 18–57%) to 64% (95% CI 43–82%) (data not shown). In both breeds, all isolates displayed solely the AmpC phenotype.

### Longitudinal Study in GPS Chickens of Breed A

Figure 1 shows, next to the ESBL/AmpC-prevalence of GPS chickens of breed A at the age of two days mentioned above, the prevalence at 18 weeks and 31 weeks. The prevalence at 18 weeks varied from 0% (95% CI 0–9%) in poultry house 1 (PH1) and poultry house 4 (PH4) to 100% (95% CI 86–100%) in poultry house 3 (PH3). The chickens in PH3 were treated twice for five days each with beta-lactam antibiotics (amoxicillin-trihydrate at two weeks of age and phenoxymethylpenicillin at eight weeks of age) which may have selected for ESBL/AmpC producing *E. coli.* In the samples taken in this poultry house, most isolates (24/25) showed an AmpC phenotype, while in PH2a and PH2b, in which the chickens were not treated with antibiotics, only ESBL-phenotypes were detected (2% and 44% positive samples, respectively) (Figure 1.2).

At 31 weeks of age (Figure 1.3), the ESBL/AmpC prevalence varied from 2% (95% CI 0–13%) in PH2 (only ESBL phenotype) to 27% (95% CI 14–43%) in PH3 (a combination of ESBL (n = 3) and AmpC (n = 8) phenotypes). AmpC types were only detected in PH3 and PH4 that obtained birds from the poultry house at the rearing farm that was treated with beta-lactam antibiotics.

The AmpC producing *E. coli* isolates found in the samples taken from the two-day-old GPS chickens were almost all found without pre-enrichment (data not shown). In contrast, the ESBL/AmpC positive samples taken at 18 weeks and 31 weeks were mostly detected by the use of a pre-enrichment step, except for the samples taken from the antibiotic treated chickens from PH3 at 18 weeks of age (data not shown).

### One-day Old PS Chickens

In the environmental samples taken from the 21 hatching units at the hatchery delivering PS chickens of broiler breed A no ESBL/AmpC producing *E. coli* were found. Two of the 649 meconium samples (0.31%) were positive for ESBL producing *E. coli* (Table 1). The PS chickens corresponding to these positive samples were derived from eggs from two different production farms. On one production farm the GPS chickens had been treated with amoxicillin (Table S1).

In all hatching units at the hatchery delivering PS chickens of broiler breed B, at least one of the environmental swabs was positive for AmpC producing *E. coli* (Table 1, one-day old PS). Nineteen of the 325 broiler PS samples (5.8%) were positive for AmpC producing *E. coli* (Table 1). The PS chickens corresponding to these samples were derived from eggs from five different production farms that were hatched in five of the six hatching units. At two production farms the GPS chickens had been treated with tylosin and on one farm with enrofloxacin (Table S1).

### One-day Old Broilers

In 12 of the 16 hatching units at least one of the environmental samples was positive for ESBL/AmpC producing *E. coli* (data not shown). Eight of the 425 broiler samples (1.9%) were positive for ESBL/AmpC producing isolates (Table 1). The chickens corresponding to these samples were derived from eggs from three different production farms and were hatched in three of the 12 hatching units in which the environment was positive for ESBL/AmpC producing *E. coli* (Table S1).

### Typing of ESBL/AmpC Genes

In isolates derived from GPS (broiler breed A and B), PS (broiler breed B) and broilers (broiler breed A) *bla*_CMY-2_ was predominantly found (Table 1). *bla*_CMY-2_ was the only ESBL/AmpC gene found in one- (or two-) day(s) old GPS chickens belonging to broiler breed A and B (Table 1 and S1). However at 18 weeks and at 31 weeks of age *bla*_TEM-52_ or *bla*_TEM-52c_ were also found in GPS chickens of broiler breed A (Table 1 and S1). These ESBL-genes were mainly found in the samples from the chickens not treated with beta-lactam antibiotics (Figure 1.2). In the isolates taken from the chickens treated with beta-lactam antibiotics (in PH3), *bla*_CMY-2_ was the dominant gene found. In the environment of the PS hatchery of broiler breed B, as in the PS chickens hatched there, only *bla*_CMY-2_ was found (Table S1). *bla*_CTX-M_ types of ESBL-genes were only found in PS chickens of broiler breed A (*bla*_CTX-M-1_ and *bla*_CTX-M-2_) and in environmental samples of hatching units at the broiler hatchery (*bla*_CTX-M-1_). Although in the chickens sampled at this hatchery only *bla*_CMY-2_ was found, in the environment the highest diversity of ESBL/AmpC genes was found: *bla*_CMY-2_, *bla*_TEM-20_, *bla*_SHV-12_ and *bla*_CTX-M-1_ and mutations in the *ampC* promotor/attenuator (*ampC* type 3) (Table S1).

### Resistance Patterns ESBL/AmpC Producing Isolates

ESBL/AmpC producing *E. coli* derived from GPS chickens from broiler breed A at two-days of age were wild-type susceptible to all non-beta-lactam antibiotics tested, however isolates from these chickens analysed at 18 weeks of age were non wild-type susceptible to the (fluoro)quinolones and one isolate was in addition non wild-type susceptible to kanamycin (Table S1). Some *E. coli* isolates collected from these animals at 31 weeks of age belonging to PH1 and PH2 were susceptible to all non-beta-lactam antibiotics and a few isolates from chickens housed in PH3 and PH4 displayed non-wild-type susceptibility to the (fluoro) quinolones (Table S1). Information from primary breeding company A revealed use of enrofloxacin for three days to prevent mortality from *E. coli* infection at arrival on the GPS rearing farm. This treatment will select for ESBL/AmpC producers that are non-susceptible to fluoroquinolones, which were indeed detected in samples taken at 18 and 31 weeks of age (Table S1). The ESBL/AmpC producing isolates collected from one-day old GPS chickens from broiler breed B that were analysed (n = 5) showed non wild-type susceptibilities to the non-beta-lactam antibiotics: nalidixic acid and ciprofloxacin (n = 1), tetracycline, streptomycin and kanamycin (n = 2), tetracycline, sulfamethoxazole, nalidixic acid, ciprofloxacin, streptomycin and kanamycin (n = 1) and one isolate was susceptible to all non-beta-lactam antibiotics tested. However the analysed isolates of broiler breed A as well as broiler breed B at PS level displayed co-non wild-type susceptibilities to non-beta-lactam antibiotics. The few ESBL-producing isolates derived from breed A displayed co-non wild-type susceptibility to gentamicin, tetracycline and sulfamethoxazole or to gentamicin, sulfamethoxazole, chloramphenicol and kanamycin (Table S1). The AmpC-producing isolates derived from breed B were mainly co-non wild-type susceptible to nalidixic acid and ciprofloxacin or susceptible to all non-beta-lactam antibiotics. At broiler level, the three out of eight ampC-producing isolates analyzed displayed either co-non wild-type susceptibility to the non-beta lactam antibiotics ciprofloxacin and nalidixic acid (n = 1), gentamicin, tetracycline, trimethoprim, ciprofloxacin and nalidixic acid (n = 1), or displayed susceptibility to all non-bet- lactam antibiotics tested (n = 1).

### Longitudinal Study at Broiler Farms

Environmental samples taken from the floor of the poultry house, before the start of the production period, contained ESBL/AmpC producing *E. coli* in two poultry houses (one out of five pools of five swabs and three out of 25 individual swabs were positive in respectively poultry house X-1 and Y-1, (Table 2). All other environmental samples taken at the broiler farms were negative (data not shown).

**Table 2:** Prevalence of broilers positive for ESBL/AmpC-producing *E. coli* at three commercial broiler farms measured in four poultry houses at arrival at the farm till week five between September 2010 and February 2011.

Poultry house	Prevalence of ESBL/AmpC-producing *E. coli* in n = 25 broilers (%, 95%CI), number of isolates with AmpC(A) or ESBL(E) phenotype					
Week	0	1	2	3	4	5
X-1*	0	100	100*	100	100	100
	(0–14)	(86–100)	(86–100)	(86–100)	(86–100)	(86–100)
		19A, 6E	15A, 9E	9A, 16E	14A, 11E	14A, 1E
Y-1*	16	100	92	100	100	100
	(4.5–36)	(86–100)	(74–99)	(86–100)	(86–100)	(86–100)
	4A	8A, 17E	15A, 8E	21A, 4E	18A, 7E	14A, 11E
Y-2	20	96	100	100	100	100
	(6.8–41)	(80–100)	(86–100)	(86–100)	(86–100)	(86–100)
	1A+E, 2A, 2E	19A, 5E	24A, 1E	24A, 1E	24A, 1E	1A+E, 17A, 7E
Z-1	4	96	100	100	100	100
	(0.1–20)	(80–100)	(86–100)	(86–100)	(86–100)	(86–100)
	1A	1A, 23E	3A, 22E	3A, 22E	9A, 16E	1A+E, 8A, 16E

* One sample was lost during processing, therefore this is 100% from 24 samples.

Feed samples taken in the four poultry houses at the start of the production period, were all negative for ESBL/AmpC producing *E. coli* (Table 3). Feed samples taken after one week were incidentally positive for ESBL/AmpC producing *E. coli* (Table 3). This was probably the result of feed contaminated by dust and manure produced by the chickens. Feed samples, from the same batch when sampled outside the poultry house, or directly from the feeding pipe inside the poultry house were negative (Table 3).

**Table 3:** Presence of ESBL/AmpC-producing isolates in broiler feed at three commercial broiler farms measured in four poultry houses at arrival at the farm till week five between September 2010 and February 2011.

Poultry house	Presence of ESBL/AmpC-producing *E. coli* in broiler feed†, AmpC(A) or ESBL(E) phenotype					
Week	0	1	2	3	4	5
X-1	−	−	+, E	−	−	−
Y-1	−	+, E	+, A	−	−	−, E
Y-2	−	−	−	−	−	+, E
Z-1	−	−	−	+, E	+, E	+, E

† ‘−’ means absence of ESBL/AmpC-producing *E. coli* in the feed sample taken in the particular poultry house in the indicated week; ‘+’ means presence of ESBL/AmpC-producing *E. coli* in the feed sample taken at the particular poultry house in the indicated week.

The three studied farms obtained their broilers from three different hatcheries. Prevalences of broilers positive for ESBL/AmpC producing *E. coli* upon arrival at poultry house X-1, Y-1, Y-2 and Z-1 were 0% (95% CI, 0–14%), 16% (95% CI 5–36%), 20% (95% CI 7–41%) and 4% (95% CI 0.1–20%), respectively. After one week the prevalences were 100% (95% CI 86–100%), 100% (95% CI 86–100%), 96% (95% CI 80–100%) and 96% (95% CI 80–100%), respectively and remained 100% (95% CI 86–100%) in all poultry houses from week three onwards (Table 2).

## Discussion

In this study we demonstrate the presence of ESBL/AmpC producing *E. coli* isolates at all levels in the broiler production pyramid. At the top of the pyramid the prevalence is lower than found in the longitudinal study at broiler production farms at the bottom of the pyramid.

The broiler production system looks very simple with only a few primary breeding companies at the top of the pyramid that produce broilers all over the world. However due to transport and trade of similar eggs and chickens to many different countries around the world, this also results in a vulnerable system. If a disease, or in this case, antibiotic resistant bacteria enter the production chain, they may be transferred globally. It is therefore worrying that ESBL/AmpC producing isolates are already present at relatively high prevalence in the top of the production pyramid.

In 2010, Sweden reported the presence of *E. coli* carrying *bla*_CMY-2_ positive strains in imported GPS chickens at their arrival in Sweden [21]. In the Netherlands, like in many other countries, the same hybrid chickens as in Sweden are used (www.sva.se). The present study confirms the Swedish findings and illustrates how these transferable resistance genes can spread in globally organized production systems.

In both broiler breeds examined, the plasmid-mediated AmpC gene *bla*_CMY-2_ was imported into the Netherlands in GPS animals derived from both the UK and Ireland or via eggs from the US. This AmpC gene was originally found in the chromosome of *Citrobacter freundii* and has spread world-wide now as a plasmid-mediated gene in many different gram-negative bacterial species. It occurs not only in isolates from poultry in Europe and the US and other parts of the world [14], [22], [23], but also in isolates derived from other animals and from humans [2], [8]. How it has entered the top of the poultry production pyramid is still unknown.

Isolates derived from broilers at five or six weeks of age, just before slaughter, can carry multiple types of ESBL/AmpC genes [14], [15], [23]. Although at the top of the production pyramid only *bla*_CMY-2_ was found, our results also show the presence of other genes (*bla*_TEM-52,_ *bla*_TEM-52c,_ *bla*_CTX-M-1_ and *bla*_CTX-M-2_) at other levels of the production pyramid, probably due to environmental contamination.

Multi-drug resistance, which is often seen in ESBL/AmpC producing isolates [24], is an important feature of these strains, because it can lead to further selection when non-beta-lactam antibiotics are used. Therefore susceptibilities to other classes of antibiotic classes were determined. The *bla*_CMY-2_ isolates from one-day old GPS birds from broiler breed A were completely susceptible to all non-beta-lactam antibiotics tested. In contrast, in the *bla*_CMY-2_-positive isolates of GPS chickens from broiler breed B co-non wild-type susceptibilities to tetracycline, streptomycin, kanamycin, sulfamethoxazole and nalidixic acid was found. Antibiotic treatment data from GGPS and pedigree stock from breed A and B showed no treatment of the particular flocks. This could indicate that the multi-resistance found in isolates from broiler breed B resulted from circulation of these isolates at the breeding farm or hatchery derived from earlier rounds.

At PS-level we again observed a difference in phenotype of the ESBL/AmpC producing *E. coli* derived from one-day old chickens of breed A (resistant to gentamicin, sulfamethoxazole, chloramphenicol and kanamycin or resistant to gentamicin, sulfamethoxazole and kanamycin) compared to the ones from breed B (sensitive or resistant to ciprofloxacin and nalidixic acid). Using one of those antibiotics can select and maintain these isolates in the production chain.

Day-old chickens can inherit bacterial isolates from their parents, or from the environment [16], [25]. In the hatching units of the PS-hatchery of breed A formaldehyde gas is used in low concentrations for infection control during hatching. Whether this may have led to the fact that no ESBL/AmpC producing isolates were found in the environmental samples of the hatching units at this hatchery, or whether this was a result of the differences in methods used (pooling five by five swabs at hatchery of breed A compared to processing individual swabs at hatchery of breed B) is not determined. It might be that a vertical transmission route through contaminated eggs [16], [18] is the most likely route of transmission in chickens of breed A. However, this would be important information for intervention measures and should be further investigated.

The observation in the longitudinal study at the broiler farms, that after the first week all broilers shed ESBL/AmpC producing *E. coli* is striking. We recently described a high prevalence of six-week-old broilers (sampled a few days before slaughter) carrying ESBL/AmpC producing *E. coli* [15]. A similar high prevalence is found on broiler meat in Dutch supermarkets [9], [10], [11]. This is reason for concern as the genes and plasmids found in isolates derived from broilers are also found in human clinical isolates [10] and indication for transmission to humans through the food chain has been documented [5].

During the first hours post hatch *E. coli* isolates (either antibiotic resistant or susceptible) are taken up by the chickens from the environment, although vertical transmission of isolates has also been described [16], [18]. Directly after hatch it takes between a few hours to a few days until *E. coli* has proliferated and has colonized the intestine of the chickens [26], [27]. Therefore analysing cloacal swabs a few hours post hatch might give variable results. A rapid increase of cloacal samples positive for ESBL/AmpC producing *E. coli* in the first week at all broiler production farms independent of antibiotic use might reflect the differences in colonization time between individual broilers. This may be the result of uptake of these isolates at the hatchery, or from feed and environmental sources at the broiler production farm [28]. No ESBL/AmpC producing *E. coli* isolates were found in the feed of the broilers during the first week, but sampling of the environment of the poultry house before the birds were placed there, resulted in the confirmation of ESBL/AmpC presence in two out of four poultry houses even after intensive cleaning and disinfection of the poultry house. This indicates that recirculation of resistant strains from earlier production rounds plays a role in contaminating the consecutive flock. The rapid increase to 100% prevalence at broiler level was in contrast to the presence of ESBL/AmpC producing isolates at GPS level. In the GPS chickens, although prevalences found were already relatively high, at 18 and 31 weeks of age no rapid increase to 100% of chickens positive for ESBL/AmpC producing *E. coli* was observed, except when treated with beta-lactam antibiotics. The results even indicated a decrease in the number of ESBL/AmpC producing isolates per sample. Compared to broilers, GPS chickens are housed at a very high level of biosecurity in which cleaning and disinfection protocols are of much higher standard and they are much less frequently treated with antibiotics (once during our study compared to multiple treatments described in broilers [15]). Feed composition differs also between GPS and broiler chickens. One of the main differences in the feed is the addition of anticoccidial compounds (nicarbazin, narasin, salinomycin or monensin) to the feed of broilers, while breeding chickens are vaccinated against coccidiosis [29]. Diarra and co-workers, demonstrated the presence of more ceftiofur resistant isolates in broilers fed with salinomycin compared to broilers fed either with non-supplemented feed or feed supplemented with bambermycin, penicillin, bacitracin or a combination of salinomycin plus bacitracin [30]. However data to explain this phenomenon were not given and this should be further investigated.

ESBL/AmpC producing isolates are found in high prevalences at broiler farms also in other European countries. In a Belgian study risk factors for ceftiofur resistance at broiler farms were determined [31]. Next to antibiotic use at the farm, management factors as well as the hatchery from where the chickens originated were risk factors for the presence of a high level of ceftiofur resistance in five week old chickens at broiler production farms [31]. This last risk factor is explained by (off-label) ceftiofur use at some broiler hatcheries in Belgium, which could have selected for these isolates. In the Netherlands, like in Belgium, third generation cephalosporins are not licensed for treatment of broilers, however also in the Netherlands up to the spring of 2010 ceftiofur has been used off-label in hatcheries [32]. The relation between the use of ceftiofur at hatcheries and the occurrence of extended spectrum cephalosporin (ESC) resistant *Salmonella* and *E. coli* in broilers has previously been described in Canada [33]. Although at the time of our longitudinal study at broiler farms, ceftiofur use at hatcheries had been stopped for almost six months [32], all broilers still became rapidly positive for ESBL/AmpC-producing *E. coli*. Whether this could be due to recirculation of these strains in the environment of the hatchery or broiler farm has to be determined. It indicates that to reduce the prevalence of ESBL/AmpC-producing isolates at broiler farms other interventions than stopping illegal antibiotic usage at the hatchery are necessary as well.

To conclude, it has been demonstrated that ESBL/AmpC producing isolates are found at every level of the broiler production pyramid. At broiler production farms these isolates spread very fast, leading to high prevalences. These high prevalences at broiler production farms are a reason for serious concern as they enter the food chain in high prevalences. Vertical transmission, horizontal transmission as well as recirculation of these isolates at farms and hatcheries may play a role. Therefore future research should not only evaluate interventions implemented at broiler farms, but should also take into account interventions implemented at hatcheries and breeding farms.

## Supporting Information

Table S1 **Results of the characterization of ESBL/AmpC genes, other resistance genes and Minimal Inhibitory Concentrations (mg/L) in isolates derived from Grandparents, Parents and broilers.**
(DOCX)
