# Supplementary material for: Presence of ESBL/AmpC -Producing Escherichia coli in the Broiler Production Pyramid: A Descriptive Study
Source: PLoS One. 2013 Nov 7;8(11):e79005. doi: 10.1371/journal.pone.0079005 (PMC3820706; doi:10.1371/journal.pone.0079005)
Supplement: Table S1 — Results of the characterization of ESBL/AmpC genes, other resistance genes and Minimal Inhibitory Concentrations (mg/L) in isolates derived from Grandparents, Parents and broilers. (DOCX) [file pone.0079005.s001.docx]

**Table S1. Results of the characterization of ESBL/AmpC genes, other resistance genes and Minimal Inhibitory Concentrations (mg/L) in isolates derived from Grandparents, Parents and broilers.**

|  |  |  |  |  |  |  |  | **MIC(mg/L)^‡^** | | | | | | | | | | | | | |
| --- | --- | --- | --- | --- | --- | --- | --- | --- | --- | --- | --- | --- | --- | --- | --- | --- | --- | --- | --- | --- | --- |
| **Broiler breed** | **Lab-code** | **Source** | **Code of production farm of origin** | **Hatching unit/poultry house no.** | **Date of sampling** | **Material** | **Results PCR and sequencing^*,†^** | **AMP** | **FOT** | **TAZ** | **GEN** | **TET** | **SMX** | **TMP** | **CIP** | **NAL** | **CHL** | **FFN** | **STR** | **KAN** | **COL** |
| A | 9 | GPS 2 days | 11-1♂ | - | 2009 Jul 8 | Caeca | *bla*_CMY-2_ | **>32** | **>4** | **16** | 0.5 | 4 | <=8 | <=0.5 | 0.03 | 8 | 16 | 16 | 4 | <=4 | <=2 |
|  | 23 | GPS 2 days | 22-1♀ | - | 2009 Jul 8 | Caeca | *bla*_CMY-2_ | **>32** | **>4** | **16** | 1 | <=1 | <=8 | <=0.5 | 0.015 | <=4 | 4 | 8 | 8 | <=4 | <=2 |
|  | 13 | GPS 2 days | 22-2♀ | - | 2009 Jul 8 | Caeca | *bla*_CMY-2_ | **>32** | **>4** | **16** | 2 | 2 | <=8 | <=0.5 | 0.015 | <=4 | 8 | 8 | 8 | <=4 | <=2 |
|  | 16 | GPS 2 days | 33-2♂ | - | 2009 Jul 8 | Caeca | *bla*_CMY-2_ | **>32** | **>4** | **16** | 1 | <=1 | <=8 | <=0.5 | 0.015 | <=4 | 4 | 4 | 4 | <=4 | <=2 |
|  | 40 | GPS 2 days | 44-2♀ | - | 2009 Jul 8 | Caeca | *bla*_CMY-2_ | **>32** | **>4** | **>16** | 1 | 4 | <=8 | <=0.5 | 0.03 | <=4 | 8 | 8 | 8 | <=4 | <=2 |
|  | 19 | GPS 2 days | 44-1♀ | - | 2009 Jul 8 | Caeca | *bla*_CMY-2_ | **>32** | **>4** | **16** | 0.5 | 4 | <=8 | 1 | 0.03 | 8 | 16 | 16 | 4 | <=4 | <=2 |
|  | 103 | GPS 18 weeks | 33-1♂+33-2♂ | 2b | 2009 Nov 9 | Cloacal swab | *bla*_TEM-52c_ | **>32** | **>4** | **8** | 1 | 2 | <=8 | <=0.5 | **0.12** | **>64** | 8 | 8 | 8 | <=4 | <=2 |
|  | 138 | GPS 18 weeks | 44-1♀ | 3 | 2009 Nov 9 | Cloacal swab | *bla*_CMY-2_ | **>32** | **>4** | **>16** | 1 | 2 | <=8 | <=0.5 | 0.03 | <=4 | 8 | 16 | 16 | <=4 | <=2 |
|  | 144 | GPS 18 weeks | 44-1♀ | 3 | 2009 Nov 9 | Cloacal swab | *bla*_TEM-52c_ | **>32** | **>4** | **4** | 1 | <=1 | <=8 | <=0.5 | **0.25** | **64** | 4 | <=2 | 8 | **16** | <=2 |
|  | 173 | GPS 18 weeks | 44-2♀ | 2a | 2009 Nov 9 | Cloacal swab | *bla*_TEM-52c_ | **>32** | **>4** | **16** | 2 | 4 | <=8 | <=0.5 | **0.5** | **>64** | 16 | 16 | 8 | <=4 | <=2 |
|  | 309 | GPS 31 weeks | 33-1♂+33-2♂+44-2♀ | 1 | 2010 Feb 8 | Cloacal swab | *bla*_TEM-52c_ | **>32** | **>4** | **8** | 1 | 4 | <=8 | <=0.5 | 0.015 | <=4 | 8 | 8 | 8 | <=4 | <=2 |
|  | 313 | GPS 31 weeks | 11-1♂+11-2♂+22-1♀+22-2♀ | 2 | 2010 Feb 8 | Cloacal swab | *bla*_TEM-52c_ | **>32** | **>4** | **8** | 1 | 2 | <=8 | <=0.5 | 0.015 | <=4 | 8 | 8 | 8 | <=4 | <=2 |
|  | 324 | GPS 31 weeks | 33-1♂+33-2♂+44-1♀+44-2♀ | 3 | 2010 Feb 8 | Cloacal swab | *bla*_CMY-2_ | **>32** | **>4** | **16** | 1 | 2 | <=8 | <=0.5 | 0.03 | <=4 | 8 | 8 | 8 | <=4 | <=2 |
|  | 325 | GPS 31 weeks | 33-1♂+33-2♂+44-1♀+44-2♀ | 3 | 2010 Feb 8 | Cloacal swab | *bla*_TEM-52_ | **>32** | **>4** | **16** | 1 | 2 | <=8 | <=0.5 | **0.25** | **>64** | 8 | 8 | 4 | <=4 | <=2 |
|  | 340 | GPS 31 weeks | 33-1♂+33-2♂+44-1♀ | 4 | 2010 Feb 8 | Cloacal swab | *bla*_CMY-2_ | **>32** | **>4** | **16** | 1 | <=1 | <=8 | <=0.5 | **0.25** | **>64** | 4 | 4 | 8 | <=4 | <=2 |
|  | 62^§^ | PS | AA♂ | 8 | 2009 Oct 6 | Meconium | *bla*_CTX-M-2_ | **>32** | **>4** | **2** | **>32** | 2 | **>1024** | <=0.5 | 0.015 | <=4 | **64** | 8 | 8 | **>128** | <=2 |
|  | 70 | PS | CC♀ | 13 | 2009 Oct 6 | Meconium | *bla*_CTX-M-1_ | **>32** | **>4** | **1** | **4** | **64** | **>1024** | <=0.5 | 0.015 | <=4 | 4 | <=2 | 4 | <=4 | <=2 |
|  | 255 | broilers | n.a. | 6 | 2010 Feb 1 | environment hatching unit | *bla*_CMY-2_ | **>32** | **>4** | **8** | 1 | <=1 | <=8 | <=0.5 | **0.25** | **>64** | 8 | 8 | 8 | <=4 | <=2 |
|  | 262 | broilers | n.a. | 7 | 2010 Feb 1 | environment hatching unit | *bla*_TEM-20_^**^ | **>32** | **>4** | **8** | **>32** | **>64** | <=8 | **>32** | **>8** | **>64** | 8 | 8 | 8 | 8 | <=2 |
|  | 264 | broilers | n.a. | 8 | 2010 Feb 1 | environment hatching unit | *bla*_CMY-2_ | **>32** | **>4** | **16** | 1 | 4 | <=8 | <=0.5 | **0.25** | **>64** | 8 | 8 | 8 | <=4 | <=2 |
|  | 237 | broilers | XXX | 11 | 2010 Feb 1 | Meconium | *bla*_CMY-2_ | **>32** | **>4** | **16** | 1 | 2 | <=8 | <=0.5 | **0.12** | **64** | 8 | 4 | 8 | <=4 | <=2 |
|  | 265 | broilers | n.a. | 11 | 2010 Feb 1 | environment hatching unit | *ampC* type 3 | **>32** | **2** | **4** | 1 | **64** | <=8 | <=0.5 | 0.03 | <=4 | 4 | 4 | 8 | <=4 | <=2 |
|  | 266 | broilers | n.a. | 11 | 2010 Feb 1 | environment hatching unit | *bla*_CMY-2_ | **>32** | **>4** | **8** | 1 | 2 | <=8 | <=0.5 | **0.12** | **64** | 8 | 4 | 8 | <=4 | <=2 |
|  | 269 | broilers | n.a. | 12 | 2010 Feb 1 | environment hatching unit | *bla*_CMY-2_ | **>32** | **>4** | **16** | 1 | 2 | <=8 | <=0.5 | **0.25** | **>64** | 8 | 8 | 4 | <=4 | <=2 |
|  | 272 | broilers | n.a. | 13 | 2010 Feb 1 | environment hatching unit | *bla*_TEM-20_*^**^* | **>32** | **4** | **1** | 1 | 2 | <=8 | <=0.5 | **0.5** | **>64** | 8 | 16 | 8 | <=4 | <=2 |
|  | 273 | broilers | n.a. | 14 | 2010 Feb 1 | environment hatching unit | *bla*_SHV-12_ | **>32** | **4** | **16** | 1 | 4 | **>1024** | **>32** | **0.5** | 16 | 8 | 8 | 8 | <=4 | <=2 |
|  | 278 | broilers | n.a. | 41 | 2010 Feb 1 | environment hatching unit | *bla*_CMY-2_ | **>32** | **>4** | **8** | 1 | 2 | <=8 | <=0.5 | 0.015 | <=4 | 8 | 8 | 8 | <=4 | <=2 |
|  | 281 | broilers | n.a. | 46 | 2010 Feb 1 | environment hatching unit | *bla*_CTX-M-1_ | **>32** | **>4** | **1** | 1 | >64 | <=8 | <=0.5 | 0.03 | <=4 | 8 | 4 | 8 | <=4 | <=2 |
|  | 243 | broilers | WWW | 47 | 2010 Feb 1 | Meconium | *bla*_CMY-2_ | **>32** | **>4** | **16** | **>32** | **>64** | <=8 | **>32** | **8** | **>64** | 8 | 8 | 8 | 8 | <=2 |
|  | 283 | broilers | n.a. | 47 | 2010 Feb 1 | environment hatching unit | *bla*_CMY-2_ | **>32** | **>4** | **16** | **>32** | **>64** | <=8 | **>32** | **>8** | **>64** | 8 | 8 | 8 | 8 | <=2 |
|  | 286 | broilers | n.a. | 47 | 2010 Feb 1 | environment hatching unit | *bla*_CTX-M-1_ | **>32** | **>4** | **2** | 1 | **>64** | **>1024** | **>32** | 0.03 | <=4 | 8 | 8 | **128** | <=4 | <=2 |
|  | 235 | broilers | ZZZ | 48 | 2010 Feb 1 | Meconium | *bla*_CMY-2_ | **>32** | **>4** | **8** | 1 | 2 | <=8 | <=0.5 | 0.015 | <=4 | 4 | 4 | 8 | <=4 | <=2 |
|  | 287 | broilers | n.a. | 48 | 2010 Feb 1 | environment hatching unit | *bla*_CMY-2_ | **>32** | **>4** | **16** | 2 | 2 | <=8 | <=0.5 | 0.015 | <=4 | 4 | 4 | 16 | 8 | <=2 |
|  | 293 | broilers | n.a. | 53 | 2010 Feb 1 | environment hatching unit | *bla*_CMY-2_ | **>32** | **>4** | **16** | 1 | 2 | <=8 | <=0.5 | 0.015 | <=4 | 8 | 4 | 8 | <=4 | <=2 |
| B | 494 | GPS | 1 | - | 2010 Nov 21 | Meconium | *bla*_CMY-2_ | **>32** | **>4** | **>16** | 1 | 2 | <=8 | <=0.5 | **0.25** | **>64** | 4 | 8 | 8 | <=4 | <=2 |
|  | 498 | GPS | 2 | - | 2010 Nov 21 | Meconium | *bla*_CMY-2_ | **>32** | **>4** | **8** | 1 | **>64** | <=8 | <=0.5 | 0.03 | <=4 | 8 | 8 | **>128** | **>128** | <=2 |
|  | 506 | GPS | 3 | - | 2010 Nov 21 | Meconium | *bla*_CMY-2_ | **>32** | **>4** | **16** | 1 | **>64** | <=8 | <=0.5 | 0.03 | <=4 | 8 | 8 | **>128** | **>128** | <=2 |
|  | 529 | GPS | 4 | - | 2010 Nov 21 | Meconium | *bla*_CMY-2_ | **>32** | **>4** | **8** | 1 | **>64** | **>1024** | <=0.5 | **0.12** | **32** | 8 | 4 | **128** | **>128** | <=2 |
|  | 531 | GPS | 5 | - | 2010 Nov 21 | Meconium | *bla*_CMY-2_ | **>32** | **>4** | **16** | 1 | 4 | <=8 | <=0.5 | 0.015 | <=4 | 4 | 4 | 4 | <=4 | <=2 |
|  | 348 | PS | n.a. | 27 | 2010 Mar 17 | environment hatching unit | *bla*_CMY-2_ | **>32** | **>4** | **16** | 1 | <=1 | **>1024** | <=0.5 | 0.015 | <=4 | 8 | 8 | 8 | <=4 | <=2 |
|  | 350 | PS | n.a. | 28 | 2010 Mar 17 | environment hatching unit | *bla*_CMY-2_ | **>32** | **>4** | **16** | 0.5 | **>64** | <=8 | <=0.5 | **0.25** | **>64** | 4 | 4 | 4 | <=4 | <=2 |
|  | 394^¶^ | PS | ZZ | 28 | 2010 Mar 17 | Meconium | *bla*_CMY-2_ | **>32** | **>4** | **16** | 1 | 2 | <=8 | <=0.5 | **0.12** | **32** | 4 | 4 | 16 | <=4 | <=2 |
|  | 354 | PS | n.a. | 29 | 2010 Mar 17 | environment hatching unit | *bla*_CMY-2_ | **>32** | **4** | **8** | 1 | 2 | <=8 | <=0.5 | 0.015 | <=4 | 8 | 8 | 8 | <=4 | <=2 |
|  | 425^¶^ | PS | SS | 29 | 2010 Mar 17 | Meconium | *bla*_CMY-2_ | **>32** | **4** | **8** | 1 | 2 | <=8 | <=0.5 | 0.015 | <=4 | 8 | 4 | 8 | <=4 | <=2 |
|  | 432^#^ | PS | VV | 29 | 2010 Mar 17 | Meconium | *ampC* type 40 | **>32** | **1** | **1** | 0.5 | 2 | <=8 | <=0.5 | **0.25** | **>64** | 8 | 16 | 4 | <=4 | <=2 |
|  | 442 | PS | UU | 29 | 2010 Mar 17 | Meconium | *bla*_CMY-2_ | **>32** | **4** | **8** | 1 | 2 | <=8 | <=0.5 | 0.015 | <=4 | 8 | 4 | 16 | <=4 | <=2 |
|  | 361 | PS | n.a. | 30 | 2010 Mar 17 | environment hatching unit | *bla*_CMY-2_ | **>32** | **>4** | **16** | 1 | <=1 | <=8 | <=0.5 | **0.5** | **>64** | 8 | 8 | 8 | <=4 | <=2 |
|  | 453^#^ | PS | VV | 30 | 2010 Mar 17 | Meconium | *ampC* type 40 | **>32** | **2** | **4** | 1 | <=1 | <=8 | <=0.5 | **0.25** | **>64** | 4 | 4 | 16 | <=4 | <=2 |
|  | 365 | PS | n.a. | 31 | 2010 Mar 17 | environment hatching unit | *bla*_CMY-2_ | **>32** | **>4** | **8** | 1 | **>64** | <=8 | <=0.5 | **0.25** | **>64** | 4 | 4 | 8 | <=4 | <=2 |
|  | 476 | PS | XX | 31 | 2010 Mar 17 | Meconium | *bla*_CMY-2_ | **>32** | **4** | **8** | 1 | 2 | <=8 | <=0.5 | **0.5** | **>64** | 4 | 4 | 8 | <=4 | <=2 |
|  | 369 | PS | n.a. | 32 | 2010 Mar 17 | environment hatching unit | *bla*_CMY-2_ | **>32** | **>4** | **16** | 1 | 4 | <=8 | <=0.5 | **0.5** | **>64** | 8 | 8 | 8 | **>128** | <=2 |
|  | 491 | PS | XX | 32 | 2010 Mar 17 | Meconium | *bla*_CMY-2_ | **>32** | **>4** | **>16** | 1 | 4 | <=8 | <=0.5 | **0.5** | **>64** | 8 | 4 | 8 | <=4 | <=2 |

*With the CTX-M-2 primers used for sequencing, no distinction can be made between *bla*_CTX-M-2_ and *bla*_CTX-M-97_.

^†^With the SHV primers used for sequencing, no distinction can be made between *bla*_SHV-12_ and *bla*_SHV-129_.

‡Bold values were considered reduced susceptible according to the EUCAST epidemiological cut-off values (www.eucast.org).

^§^Isolates that were derived from offspring of breeding chickens treated with amoxicillin.

^¶^Isolates that were derived from offspring of breeding chickens treated with tylosin.

^#^Isolates that were derived from offspring of breeding chickens treated with enrofloxacin.

^**^*bla*_TEM-20_ with silent mutations +144G->A, +480C->T and +723A->G compared to reference sequence *bla*_TEM-20_ Y17581 (nucleotide position according to amino acid count at www.lahey/studies.org)
